# EIF2S1 Silencing Impedes Neuroblastoma Development Through GPX4 Inactivation and Ferroptosis Induction

**DOI:** 10.1155/2024/6594426

**Published:** 2024-10-19

**Authors:** Zhen Li, Yunhui Wang, Shubin Liang, Tingdong Yuan, Jing Liu

**Affiliations:** ^1^Department of General & Pediatric Surgery, Yantai Yuhuangding Hospital, No. 20 Yuhuangding East Road, Zhifu District, Yantai 264099, China; ^2^Department of Pathology, Yantai Yuhuangding Hospital, No. 20 Yuhuangding East Road, Zhifu District, Yantai 264099, China

**Keywords:** Fe^2+^, ferroptosis, neuroblastoma, reactive oxygen species

## Abstract

**Background:** Neuroblastoma (NB) is one of the most devastating malignancies in children, accounting for a high mortality rate due to limited treatment options. This study is aimed at elucidating the role of the ferroptosis-related EIF2S1 gene in NB pathogenesis and exploring its potential as a therapeutic target.

**Methods:** We conducted comprehensive bioinformatics analyses utilizing the FerrDb database and NB-related transcriptomics data to investigate the role of EIF2S1 in NB. Changes in EIF2S1 expression were subsequently validated in NB tissues and cell lines. Loss-of-function experiments were performed in SK-N-SH and IMR-32 cell lines through shRNA-mediated EIF2S1 knockdown. The impact of EIF2S1 knockdown on the tumorigenesis of SK-N-SH cells was assessed in nude mice.

**Results:** Bioinformatics analyses revealed a significant association between elevated EIF2S1 expression and poor prognosis in NB patients. The increased levels of EIF2S1 expression were confirmed in NB tissues and cancerous cell lines. Furthermore, EIF2S1 overexpression was linked to translational regulation and immune cell infiltration modulation. Silencing of EIF2S1 resulted in the suppression of cell proliferation, migration, and tumorigenicity in NB cells. Additionally, EIF2S1 knockdown led to an accumulation of iron and oxidative stress, as well as a reduction in GPX4 and SLC7A11 expression.

**Conclusion:** Our findings indicate that EIF2S1 appears to facilitate the progression of NB by protecting tumor cells from ferroptosis through modulating GPX4 and SLC7A11 expression. Consequently, EIF2S1 may serve as a potential therapeutic target for the management of NB.

## 1. Introduction

Neuroblastoma (NB) is classified as an embryonal neoplasm that originates from the undifferentiated sympathetic ganglion cells within the neural crest [1]. It is the most common extracranial solid tumor in children, with a particularly high incidence in the 1–5-year-old age group, earning it the moniker “king of childhood tumors” [2]. Numerous reports indicate that the increased fatality rate associated with this condition is linked to several factors, including an inconspicuous site of origin, the absence of distinctive symptoms, challenges in early diagnosis, and a tendency for multiple metastatic occurrences [3]. Current treatment modalities encompass adjuvant therapies such as autograft transplantation and radiation, along with chemotherapeutic interventions; however, most of these approaches demonstrate limited efficacy [4]. Therefore, a thorough investigation of the biochemical characteristics and underlying mechanisms of NB is imperative to facilitate advancements in therapeutic strategies.

Ferroptosis, a distinct form of iron-dependent programmed cell death, has emerged as a promising target in cancer therapy [5]. Unlike apoptosis, a well-studied form of programmed cell death characterized by cell shrinkage, activation of caspase enzymes, chromatin condensation, DNA fragmentation, and the formation of apoptotic bodies, ferroptosis is triggered by iron-dependent lipid peroxidation [5–7]. An excess of intracellular iron promotes Fenton chemistry and leads to the accumulation of reactive oxygen species (ROS), and the deadly ROS attack on lipids leads to lipid peroxidation and the disintegration of the cell membrane in ferroptosis [6]. This process increases membrane permeability, resulting in the loss of intracellular constituents and eventual cell death [7]. Ferroptosis is morphologically and biochemically distinct from apoptosis, lacking typical apoptotic features such as caspase activation or apoptotic body formation [5–7]. Tumor cells, characterized by metabolic reprogramming, tend to possess elevated levels of iron and ROS, rendering them more susceptible to ferroptosis compared to normal cells [8–10]. Recent evidence has indicated that ferroptosis-related gene signatures are intricately associated with clinical outcomes in NB [11]. For instance, activation of oncogenic MYCN in NB cells leads to the augmentation of antioxidant capacity, shielding NB cells from ferroptosis [12]. Conversely, silencing ferroportin, the sole iron-exporting protein, amplifies ferroptotic responses in NB cells [13]. Despite these insights, the factors involved in regulating ferroptosis susceptibility in NB remain to be fully elucidated.

In this study, we sought to identify critical ferroptosis-associated genes in NB by analyzing target NB RNAseq data. Through univariate and multivariate analyses, we identified EIF2S1 (Eukaryotic Translation Initiation Factor 2 subunit alpha) as a ferroptosis-related gene potentially related to NB prognosis. EIF2S1 plays a pivotal role in the initial regulated step of protein synthesis, facilitating the binding of initiator tRNA to 40S ribosomal subunits [14]. A recent bioinformatics study also reported EIF2S1 as a negative prognostic factor in NB [15]. However, the biological roles and the involvement of elevated EIF2S1 expression in ferroptosis sensitivity have not been studied. To address this knowledge gap, we validated EIF2S1 expression levels in NB tissues and cell lines and conducted loss-of-function experiments in vitro and in vivo. Particularly, the impact of EIF2S1 on ferroptosis regulation was explored. Our findings suggest that EIF2S1 protects NB cells from ferroptosis by upregulating GPX4 and SLC7A11 expression, highlighting its potential as a therapeutic target for NB treatment.

## 2. Methods

### 2.1. Bioinformatics Analyses

The FerrDb V2 database (http://www.zhounan.org/ferrdb/current/) was utilized to identify ferroptosis-related genes, leading to the identification of 382 genes, which include gene drivers, markers, and suppressors. Additionally, gene expression data were obtained from the NB RNAseq dataset available in the target database (https://ocg.cancer.gov/programs/target), which was integrated with clinical information. Subsequently, univariate and multivariate Cox regression analyses were performed to elucidate the key genes associated with NB prognosis.

### 2.2. Correlation and Enrichment Analyses

A Pearson correlation analysis was conducted using the transcriptomics data from NB to examine the relationship between EIF2S1 and the expression levels of other mRNAs. Subsequently, the genes that exhibited a positive correlation coefficient with EIF2S1 were subjected to enrichment analysis. Functional assessment was performed using gene ontology (GO) enrichment analysis (enrichGO) and the Kyoto Encyclopedia of Genes and Genomes (KEGG) within the clusterProfiler framework, with a significance threshold set at *p* < 0.05 to identify significant enrichment.

### 2.3. Tissue Specimens

During the procedure for surgical resection, tissue samples of NB (*n* = 36) were collected from individuals who had recently been diagnosed with NB. Detailed baseline data, including survival duration, were carefully gathered. Furthermore, the tumors that were resected underwent pathological classification in accordance with the International Neuroblastoma Staging System (INSS), and fluorescence in situ hybridization (FISH) tests were performed clinically to identify gene amplification. For comparison purposes, samples of normal human dorsal root ganglia (*n* = 15) obtained from terminated pregnancies were employed as a negative control. Informed consent forms were completed and signed by the patients' parents or legal guardians. The study was approved by the Medical Ethics Committee of Yantai Yuhuangding Hospital (2018112501).

### 2.4. Cell Culture

Human umbilical vein endothelial cells (HUVECs) were sourced from Procell Life Science & Technology Co. Ltd. (Wuhan, China). These cells were grown in Roswell Park Memorial Institute (RPMI1640) medium (Thermo Fisher, Waltham, United States) with 10% fetal bovine serum (FBS, Cat. # SV30160.03, GE Healthcare Life Science, Shanghai, China). Various NB cell lines, including SK-N-SH, SK-N-AS, SK-N-BE(2), and IMR-32, were acquired from the American Type Culture Collection (ATCC, Manassas, United States) and were cultured in Minimum Essential Medium (MEM, Thermo Fisher) with 10% FBS. Cell culture was maintained in standard culture conditions at 37°C with a 5% CO_2_ atmosphere.

### 2.5. Short Hairpin Ribose Nucleic Acid (shRNA)-Mediated Gene Knockdown

Three different shRNAs targeting EIF2S1 (shRNA1, shRNA2, and shRNA3), along with the corresponding shRNA negative control (shRNA-NC), were synthesized by Tianyi Huayu Biological Technology Co. Ltd. (Wuhan, China): shRNA1#, 5⁣′-TTTCCTGAGGTGGAAGATGTA-3⁣′; shRNA2#, 5⁣′-TCCGTTCTATCAACAAACTCA-3⁣′; and shRNA3#, 5⁣′-CTCATCCGAATTGGCAGGAAT-3⁣′. These sense and antisense single-stranded oligonucleotides were subsequently subjected to an annealing procedure. The lentiviral plasmids expressing these shRNAs were constructed by digesting the shRNA oligonucleotides and ligating them into pLKO.1-puro vectors (Sigma-Aldrich, St. Louis, United States). Subsequently, the resulting pLKO.1-puro vectors containing the shRNAs were packaged using MISSION Lentiviral Packaging Mix (Sigma-Aldrich, Shangai, China) in 293 T cells through the CaCl_2_ transfection method. Lentiviral particles were collected 48 h posttransfection. NB cells were transduced with lentiviral particles at a multiplicity of infection (MOI) of 60 for 48 h, followed by the selection with 800 ng/mL puromycin for 10 days to generate stable cell clones. The efficiency of EIF2S1 knockdown was subsequently validated using the Western blot analysis.

### 2.6. RT-qPCR Analysis

Total RNA samples were extracted from the cells or tissues using TRIzol reagent (Cat#15596026, Thermo Fisher). Following the verification of RNA purity and integrity, complementary DNA (cDNA) was synthesized from the RNA samples using oligo primers and RT Master Mix for qPCR II (MedChemExpress, China). The synthesized cDNA served as the template for real-time PCR analysis conducted on the HT7900 system (ABI system). Finally, data analysis was performed using the 2^−ΔΔCt^ method using GAPDH as the internal reference gene. The following primer sequences were used in RT-qPCR analysis:

• EIF2S1:   Forward: 5⁣′-GGGGAAGCAAGTCTGGTCTC-3⁣′.

      Reverse: 5⁣′-CAAGCTGACATAAGCCCCCA-3⁣′.

• GAPDH:    Forward: 5⁣′-GGAGCGAGATCCCTCCAAAAT-3⁣′.

      Reverse: 5⁣′-GGCTGTTGTCATACTTCTCATGG-3⁣′.

### 2.7. Western Blot Analysis

Cells were initially subjected to a rinse with phosphate-buffered saline (PBS) at ice-cold temperatures, followed by a treatment with ribonucleoprotein immunoprecipitation (RIP) lysis buffer (Beyotime, Beijing, China). Protein quantification was then carried out using a bicinchoninic acid (BCA) assay quantification kit (Bio-Rad, Hercules, United States). Next, 20 *μ*g of protein was loaded into each lane of a 10% sodium dodecyl sulfate–polyacrylamide gel for electrophoresis. The separated protein bands were subsequently transferred from the gel to polyvinylidene fluoride (PVDF) membranes utilizing the iBlot system (Invitrogen, Waltham, United States). These membranes were subsequently placed in an ice-cold buffer containing primary antibodies and left to incubate overnight, including SLC7A11 (Cat#K009230P, Solarbio Life Sciences, Beijing, China), EIF2S1 (Cat# K010094P, Solarbio), GPX4 (Cat#K003083P, Solarbio), N-Myc (Cat#ab24193, Abcam), and antitubulin (Cat#K200059M, Solarbio). Further, the membranes were incubated with secondary antibodies (Cat#A-11008 or A-11001, Thermo Fisher) for 2 h at room temperature. Protein band visualization was achieved using an enhanced chemiluminescence (ECL) detection system (Pierce, Rockford, IL, United States).

### 2.8. Cell Counting Kit (CCK)-8 Assay

NB cells (SK-N-SH and IMR-32 cell lines) were plated at a density of 3500 cells per well in 96-well plates. The cells were allowed to grow for varying durations from 24 to 96 h, respectively. Following these incubation periods, 10 *μ*L of CCK-8 reagent (Catalog#HY-K0301, MCE, MedChemExpress) was introduced to each well, and incubation continued for an additional 3 h. Finally, the optical density (OD) readings of each well at 460 nm were recorded using a BioTek microplate reader (BioTek microplate reader) to evaluate cell proliferation capabilities.

### 2.9. Transwell Migration and Invasion Assay

The transwell inserts (with 8-*μ*m pores, Corning Inc., Corning, United States) were inoculated with 2 × 10^5^ cells in 300 *μ*L of FBS-free media. The inserts were precoated with Matrigel (Corning) for the invasion assay, while uncoated inserts were utilized in the migration assay. The inserts were then placed in the lower chambers of a 24-well plate containing 350 *μ*L culture medium. Twenty percent of FBS was added to the lower chambers as the attractant. After a 24-h incubation period, nonmigrating/invading cells on the upper surfaces of the inserts were gently removed, and the cells that had migrated or invaded to the lower side of the inserts were fixed in 70% methanol for a duration of 10 min. Following fixation, these cells underwent a staining process with 0.1% crystal violet (Catalog Number: C0121 from Beyotime Biotechnology Co. Ltd., China) for an additional 10 min. Cells were counted under a light inverted microscope (model Olympus 1X71, manufactured by Olympus Corporation) at 200× magnification. Migrating/invading cells in five random fields were counted for each sample.

### 2.10. TUNEL Assay

Initially, 3 × 10^6^ SK-N-SH or IMR-32 cells were plated in 12-well plates and subsequently fixed with 1% paraformaldehyde (Cat#16005 Sigma-Aldrich) for 15 min. Following this, cell membrane permeability was enhanced by incubating with 0.1% Triton X-100 for 5 min. Next, the cells were treated with a TUNEL reaction solution from the TUNEL fluorescence labeling kit (C1086, Beyotime) for 1 h at room temperature, shielded from light. The cell nuclei were counterstained with 4⁣′,6-diamidino-2-phenylindole (DAPI, Sigma-Aldrich) for 10 min at room temperature. Cells from five random microscopic fields were examined for TUNEL signals under a fluorescence microscope (Olympus 1X71; Olympus Corporation).

### 2.11. Measurement of ROS, Malondialdehyde (MDA), and Fe^2+^ Levels

The levels of ROS generated intracellularly, as well as MDA and Fe^2+^, were measured utilizing commercial kits based on the supplier's instructions, including the ROS assay (Cat# R252, Dojindo Molecular Technologies Inc., Beijing, China), the MDA assay (Cat# M496, Dojindo Molecular Technologies Inc.), and the iron assay (Cat# I291, Dojindo Molecular Technologies Inc.). Data were normalized against the protein content in each sample for comparisons.

### 2.12. Xenograft Tumor Assay

BALB/c-nu mice (*n* = 12, aged 6–8 weeks, and weighing 23 ± 2 g) were purchased from Shanghai SLAC Laboratory Animal Co. Ltd. (Shanghai, China). The mice were kept in a controlled animal facility where the temperature was regulated between 22°C and 24°C, with humidity levels sustained at 55%–60%, a light-dark cycle of 12 h, and unhindered access to food and water. The experiments involving animals received approval from the Animal Care and Use Committee of Yantai Yuhuangding Hospital (2022DL055), and all animal experimental procedures complied with the NIH Guide for the Care and Use of Laboratory Animals, in addition to meeting the requirements of the ARRIVE guidelines. Xenograft tumors were established by subcutaneously implanting 2 × 10^6^ SK-N-SH cells per animal at the right flank. The mice were inoculated with cells expressing either shRNA-EIF2S1#1 or shRNA-NC (*n* = 5 per group). Tumor size was measured weekly, and all the animals were euthanized using CO_2_ asphyxiation 4 weeks after tumor cell inoculation. Tumor samples were immediately collected from terminally dead mice and subjected to Ki67 and EIF2S1 immunohistochemistry analysis.

### 2.13. Statistical Analysis

The data were summarized as mean ± standard deviation (SD) using GraphPad Prism 8.0 (GraphPad Software, New York, United States). Unpaired *t*-tests or one-way analysis of variance (ANOVA) were employed to assess statistical differences between groups, depending on whether there were two or more groups. Results yielding a *p* value of less than 0.05 were regarded as statistically significant.

## 3. Results

### 3.1. EIF2S1 Is Highly Expressed in NB Tissues

**Figure 1:**
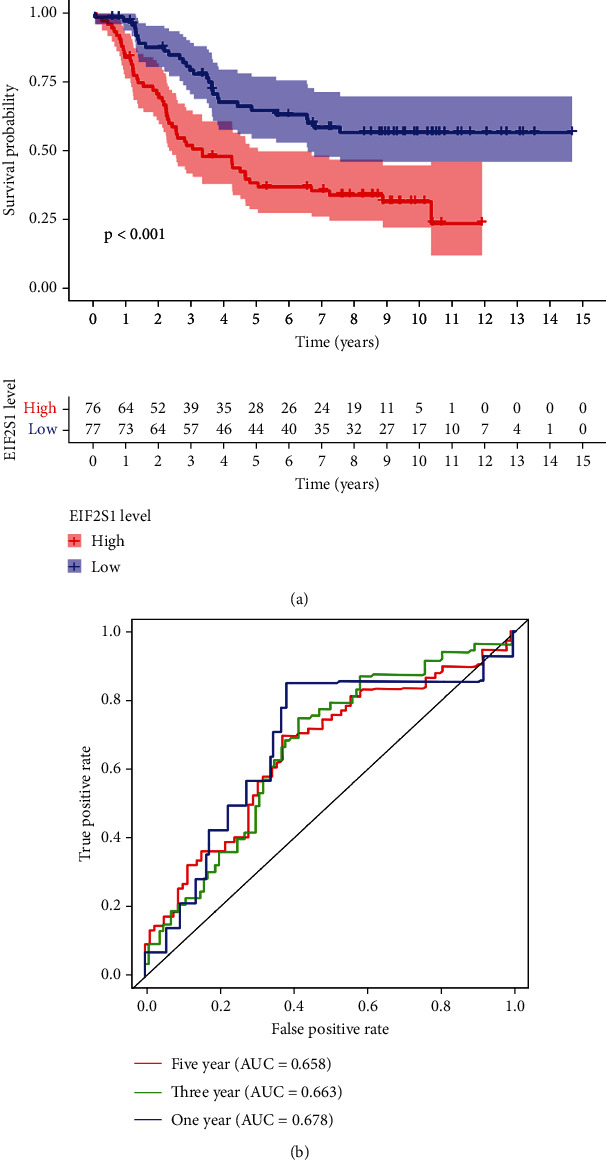
EIF2S1 expression is associated with poor prognosis in neuroblastoma. (a) Kaplan–Meier survival analysis of neuroblastoma patients from the TCGA database stratified by high- and low-EIF2S1 expression. Patients with high EIF2S1 expression showed significantly poorer overall survival (*p* < 0.001). (b) Receiver operating characteristic (ROC) curve analysis demonstrating the diagnostic value of EIF2S1 in neuroblastoma. The area under the curve (AUC) values were 0.678, 0.663, and 0.658 for 1-, 3-, and 5-year survival predictions, respectively.

Given the high susceptibility of NB cells to ferroptosis [12], we initially employed the FerrDb database (http://www.zhounan.org/ferrdb/current/) to explore various ferroptosis-associated genes. We discovered a total of 382 genes, including ferroptosis promoters, suppressors, and biomarkers. Subsequent univariate and multivariate analyses revealed that six ferroptosis-associated genes, including AKR1C1, EIF2S1, FTH1, HSF1, LONP1, and ULK2, were associated with inferior prognosis (Table 1). Among these, EIF2S1 displayed the highest hazard regression (HR), indicating its association with the highest risk for poor prognosis of NB. We further corroborated the prognostic significance of the EIF2S1 gene using a Kaplan–Meier (KM) plotter-based survival analysis of The Cancer Genome Atlas (TCGA)-NB database. Results showed that NB patients with elevated EIF2S1 expression exhibited markedly poorer prognostic outcomes compared to those with low EIF2S1 expression (Figure 1(a), *p* < 0.001). In the TCGA-NB cohort, EIF2S1 also demonstrated significant diagnostic value, with AUC values of 0.678 for 1 year, 0.663 for 3 years, and 0.658 for 5 years (Figure 1(b)).

**Figure 2:**
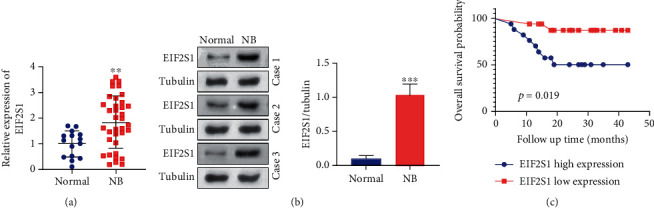
EIF2S1 is upregulated in neuroblastoma tissues and correlates with poor clinical outcomes. (a) RT-qPCR analysis showing significantly higher EIF2S1 mRNA levels in neuroblastoma tissues compared to normal tissues (*p* < 0.001, *n* = 36). (b) Western blot analysis demonstrating increased EIF2S1 protein expression in neuroblastoma tissues compared to normal tissues. (c) Kaplan–Meier survival analysis of neuroblastoma patients based on EIF2S1 protein expression levels. Patients with high EIF2S1 expression exhibited significantly poorer overall survival compared to those with low expression (*p* = 0.019).

To validate these bioinformatics findings, we evaluated the significance of the EIF2S1 gene in NB tissue samples (*n* = 36) from clinical NB cases. NB tissues exhibited significantly higher EIF2S1 mRNA levels compared to tumor-free tissues from healthy counterparts (Figure 2(a), *p* < 0.001). Western blot analysis further corroborated these findings, revealing upregulation of EIF2S1 protein in NB tissues compared to normal tissues (Figure 2(b)). We also analyzed the survival data of enrolled NB patients based on EIF2S1 protein expression. Consistent with our previous findings, patients with high EIF2S1 expression exhibited poorer overall survival rates compared to those with low EIF2S1 expression (Figure 2(c), *p* = 0.019). Chi-square test results comparing clinicopathological demographics between high and low-EIF2S1-expressing NB patients showed that upregulation of EIF2S1 was positively associated with tumor stage, risk of NB, and incidence of distant metastasis (Table 2). Collectively, these results indicate that high EIF2S1 expression is a predominant feature of NB patients, which is associated with dismal disease progression.

### 3.2. Enrichment Analysis of EIF2S1-Related Biological Processes and Pathways

**Figure 3:**
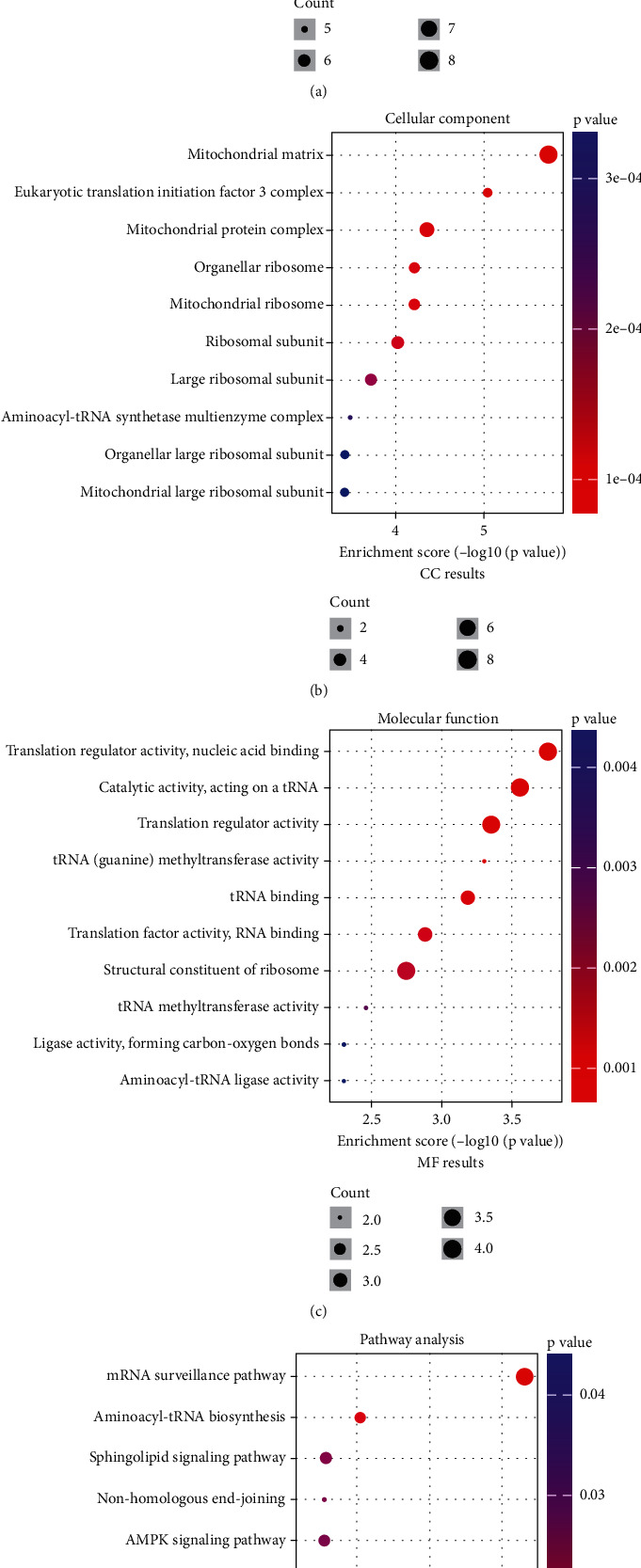
Enrichment analysis of EIF2S1-related biological processes and pathways. (a) Gene ontology (GO) analysis of biological processes associated with EIF2S1 expression. (b) GO analysis of cellular components associated with EIF2S1 expression. (c) GO analysis of molecular functions associated with EIF2S1 expression. (d) KEGG pathway enrichment analysis of processes associated with EIF2S1 expression in TCGA-NB cohort.

To investigate the potential mechanisms underlying EIF2S1 functionality, we undertook a comprehensive series of correlation and enrichment analyses based on the TCGA-NB database. Pearson's analysis unveiled 50 mRNAs exhibiting significant correlation with EIF2S1 expression (correlation coefficient of > 0.6, *p* < 0.001; Supporting Information 1 and 2). We included the most positively EIF2S1-associated genes for analyzing GO function and KEGG pathway enrichment (Figures 3(a), 3(b), 3(c), and 3(d)). The GO-based biological process (GO-BP) analysis revealed positive correlations between EIF2S1 and processes including translational termination, mitochondrial gene expression, mitochondrial translation, cellular protein complex disassembly, and noncoding RNA (ncRNA) metabolic processes. The GO cellular component (GO-CC) analysis showed positive correlations between EIF2S1 and CCs such as mitochondrial matrix, Eukaryotic Translation Initiation Factor 3 complex, and mitochondrial protein complex. The GO molecular function (GO-MF) analysis indicated positive associations between EIF2S1 and functions including translation regulator activity, nucleic acid binding, and catalytic activity acting on tRNA. KEGG pathway enrichment analysis highlighted predominant enrichment in pathways including mRNA surveillance, aminoacyl-tRNA biosynthesis, sphingolipid signaling, nonhomologous end-joining, AMPK signaling, and mismatch repair. Collectively, these findings point to the involvement of EIF2S1 in a multitude of biological processes and pathways within NB cells.

### 3.3. Functional Differences Between High- and Low-EIF2S1 Expression Groups Based on GO and KEGG Pathway Analyses

**Figure 4:**
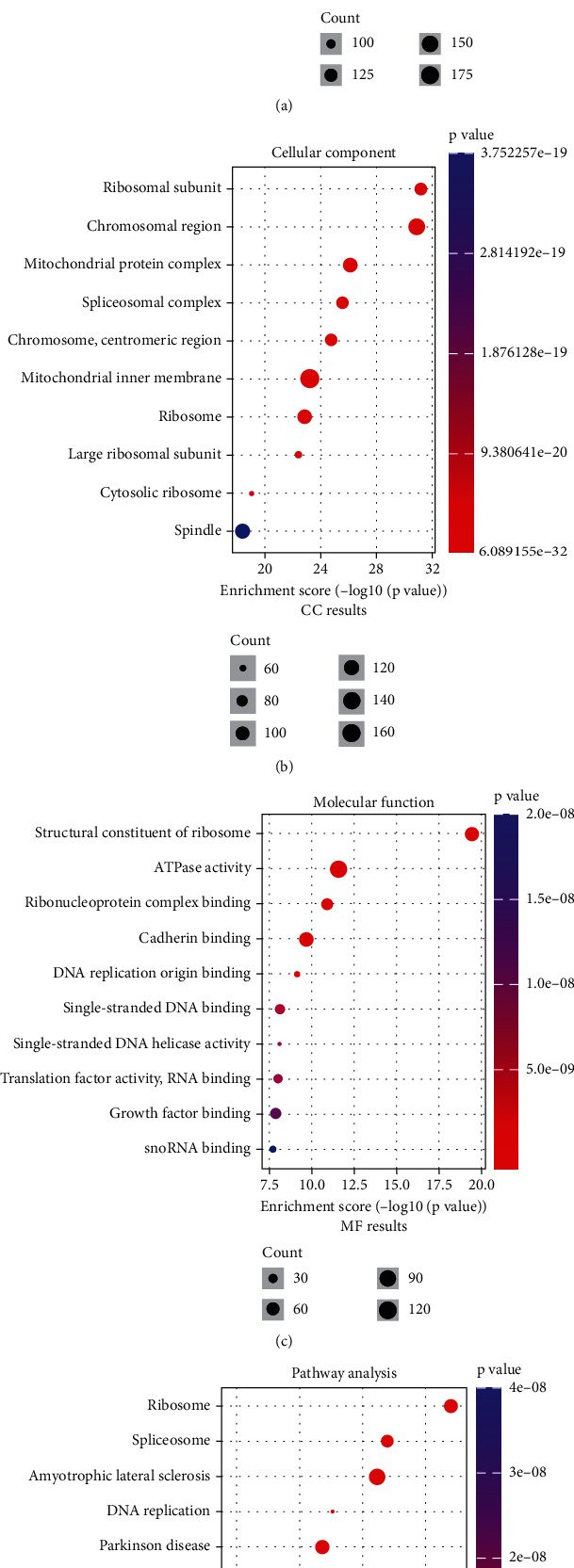
Functional differences between high- and low-EIF2S1 expression groups based on GO and KEGG pathway analyses. (a) Gene ontology (GO) analysis of biological processes, (b) cellular components (CC), (c) molecular functions (MF), and (d) KEGG pathway enriched in the differentially expressed genes between high- and low-EIF2S1 expression samples from the GSE49710 dataset.

We further explored the disparities in enrichment analyses between high- and low-EIF2S1 expression groups. We identified 4316 differentially expressed genes (DEGs) between the two groups (LogFC > 1, *p* < 0.05; Supporting Information 3). GO and KEGG enrichment analyses of these DEGs revealed that genes associated with EIF2S1 were predominantly enriched in biological processes such as ribonucleoprotein complex biogenesis, ribosome biogenesis, viral gene expression, and RNA splicing (Figures 4(a), 4(b), 4(c), and 4(d)). In CC categories, EIF2S1-associated DEGs are correlated with ribosomal subunit, chromosomal region, mitochondrial protein complex, and spliceosomal complex. MF categories showed enrichment in structural constituents of the ribosome, ATPase activity, ribonucleoprotein complex binding, and DNA replication origin binding. KEGG analysis substantiated the enrichment of pathways shared between both groups, including ribosome, spliceosome, DNA replication, cell cycle, and oxidative phosphorylation (Figure 4).

### 3.4. Comparison of Immune Activity Between High- and Low-EIF2S1 Groups

**Figure 5:**
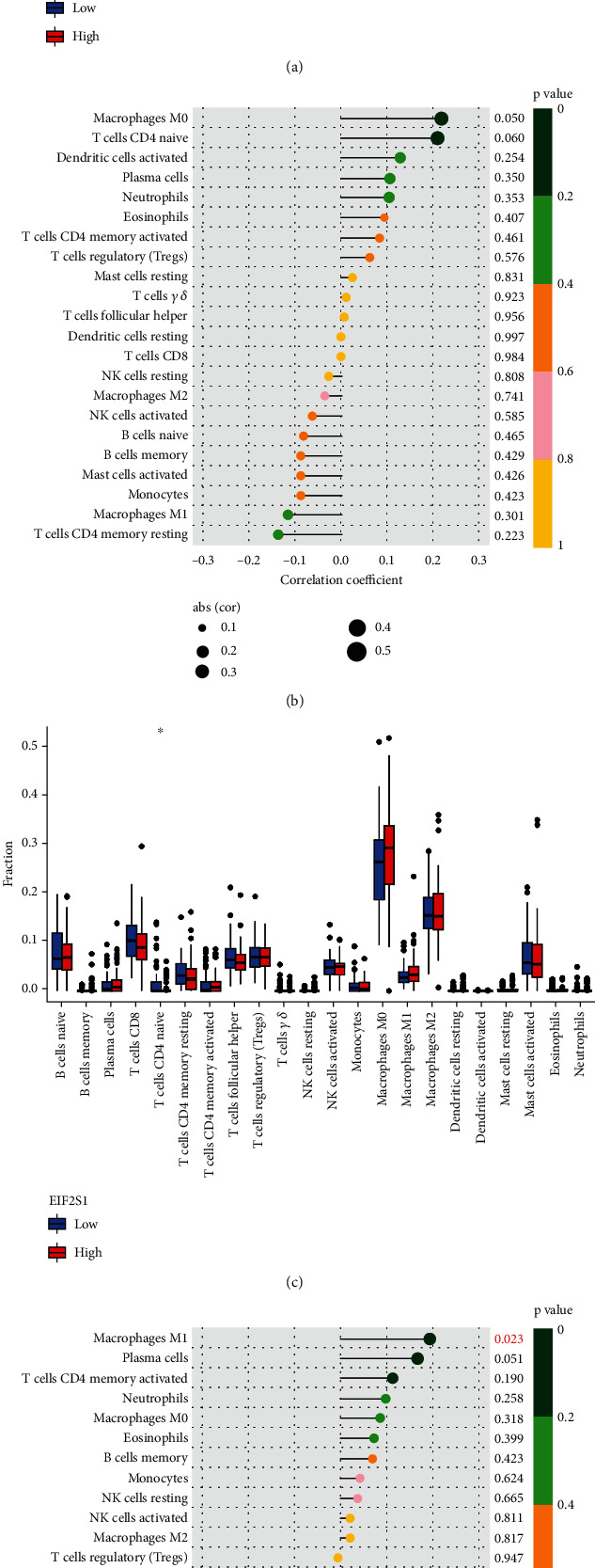
Correlation between immune cell infiltration and EIF2S1 expression. (a) Immune cell infiltration analysis in the EIF2S1-high and EIF2S1-low expression groups in the TCGA-NB cohort. (b) The summary of correlations of different immune cells with EIF2S1 expression in TCGA-NB cohort. (c) Immune cell infiltration analysis in the EIF2S1-high and EIF2S1-low expression samples from the GSE49710 dataset. (d) The summary of correlations of different immune cells with EIF2S1 expression in the GSE49710 dataset.

Given that EIF2S1 may modulate immune activation [16], we explored its role in immune cell infiltration during NB progression. Analysis of the TCGA-NB cohort indicated a higher abundance of macrophage M0 cells in the high-EIF2S1 group compared to the low-EIF2S1 group (Figures 5(a) and 5(b)). Furthermore, immune infiltration patterns from the GEO database (GSE49710) demonstrated that increased EIF2S1 mRNA levels were associated with higher levels of macrophage M1 cells, naive CD T-cells, and follicular helper T-cells (Figures 5(c) and 5(d)). These findings collectively support a potential immunomodulatory role for EIF2S1 in NB progression.

### 3.5. EIF2S1 Silencing Impedes NB Tumor Growth In Vitro and In Vivo

**Figure 6:**
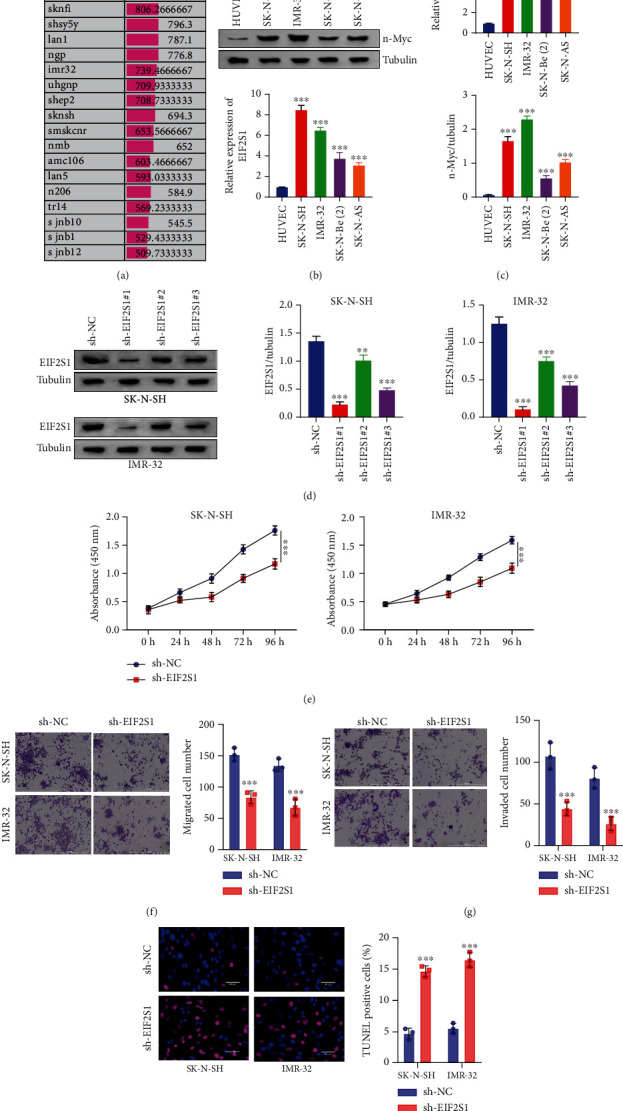
EIF2S1 silencing attenuates the malignancy of neuroblastoma cells. (a) Relative expression of EIF2S1 mRNA in various neuroblastoma cell lines from the GSE28019. (b) RT-qPCR analysis of EIF2S1 mRNA levels in SK-N-SH, IMR-32, SK-N-BE(2), and SK-N-AS cells compared to control HUVECs. (c) RT-qPCR and Western blot analysis of N-Myc expression levels in the above cell lines. (d) Western blot analysis of EIF2S1 protein levels in SK-N-SH and IMR-32 cells transduced with lentivirus carrying sh-EIF2S1#1, sh-EIF2S1#2, sh-EIF2S1#3, or sh-NC. (e) Cell proliferation assessed by CCK-8 assay in SK-N-SH and IMR-32 cells transduced with lentivrus carrying sh-EIF2S1#1 or sh-NC. (f) Transwell migration assay results for SK-N-SH and IMR-32 cells transduced with lentivrus carrying sh-EIF2S1#1 or sh-NC. (g) Transwell invasion assay results for SK-N-SH and IMR-32 cells transduced with lentivrus carrying sh-EIF2S1#1 or sh-NC. (h) TUNEL staining results showing apoptotic cells in SK-N-SH and IMR-32 cells transduced with lentivrus carrying sh-EIF2S1#1 or sh-NC. *sh-EIF2S1* versus *sh-NC*; ⁣^∗∗∗^ indicates *p* < 0.0001.

To investigate the biological function of EIF2S1 in NB cells, we first analyzed the relative expression of EIF2S1 in various NB cell lines using the GSE28019 database, which revealed high expression levels of EIF2S1 mRNA across multiple NB cell lines (Figure 6(a)). RT-qPCR analysis confirmed the elevated EIF2S1 mRNA levels in SK-N-SH, IMR-32, SK-N-BE(2), and SK-N-AS cells compared to control HUVECs (Figure 6(b), *p* < 0.05). These NB cell lines also exhibited an overexpression of N-Myc proteins (Figure 6(c)), which is a characteristic of NB due to *MYCN* gene amplification [4]. Based on these results, we selected SK-N-SH and IMR-32 cells for further experiments due to their higher EIF2S1 mRNA expression. Next, sh-EIF2S1#1, sh-EIF2S1#2, and sh-EIF2S1#3 were introduced into the selected NB cell lines to construct EIF2S1-silenced NB cells. Western blot analysis confirmed the silencing effects of these shRNAs compared to the sh-NC control (Figure 6(d)). Given the maximum silencing efficiency of sh-EIF2S1#1, NB cells stably expressing sh-EIF2S1#1 were used for subsequent in vitro functional assays. The CCK-8 assay demonstrated that NB cell proliferation was significantly reduced after EIF2S1 silencing (Figure 6(e), *p* < 0.0001). Transwell assay revealed impaired cell migration and invasion upon EIF2S1 silencing in both NB cell lines (Figures 6(f) and 6(g), *p* < 0.0001). Furthermore, TUNEL staining revealed a higher percentage of apoptotic cells in sh-EIF2S1 NB groups, indicating a higher degree of cell death after EIF2S1 silencing compared to the sh-NC treatment group (Figure 6(h), *p* < 0.0001).

**Figure 7:**
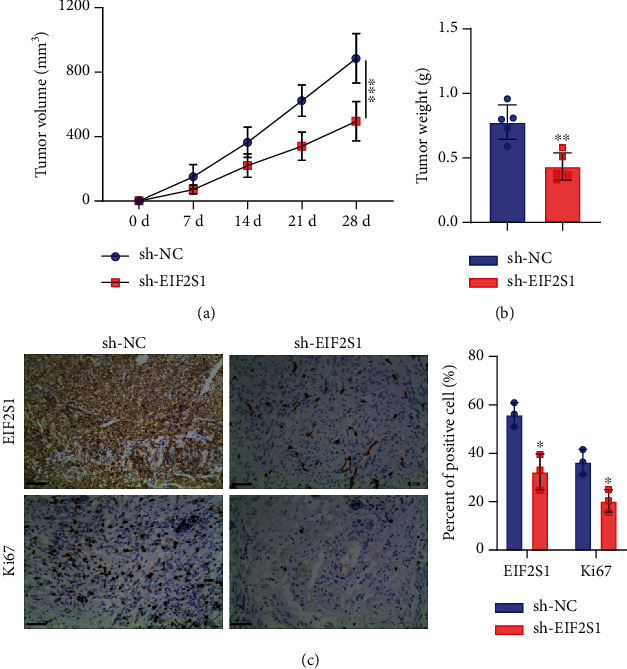
EIF2S1 silencing impedes neuroblastoma tumor growth in vivo. (a) Tumor size measurements of xenograft tumors derived from SK-N-SH cells expressing sh-EIF2S1 or sh-NC. (b) Tumor weights of xenograft tumors derived from SK-N-SH cells expressing sh-EIF2S1 or sh-NC. (c) Immunohistochemical staining and quantification of Ki67-positive cells in tumor tissues from sh-EIF2S1 and sh-NC groups. *sh-EIF2S1* versus *sh-NC*; ⁣^∗^ indicates *p* < 0.05, ⁣^∗∗^ presents *p* < 0.001, and ⁣^∗∗∗^ signifies *p* < 0.0001.

To further evaluate the impact of EIF2S1 on NB tumorigenesis in vivo, we generated xenograft tumor models using SK-N-SH cells expressing sh-EIF2S1 or sh-NC, since SK-N-SH cells exhibited the highest level of EIF2S1 among all NB cell lines. Measurements of tumor size and weights indicated a reduced tumor burden in the sh-EIF2S1 group (Figures 7(a) and 7(b), *p* < 0.0001, *p* < 0.001). Immunohistochemical staining in the tumor tissues showed a significant decrease of Ki67-positive cells in the samples with EIF2S1 silencing, further indicating the impeded cell proliferation in vivo (Figure 7(c), *p* < 0.05).

### 3.6. EIF2S1 Silencing Increases Cellular ROS and Induces Ferroptosis Features in NB Cells

**Figure 8:**
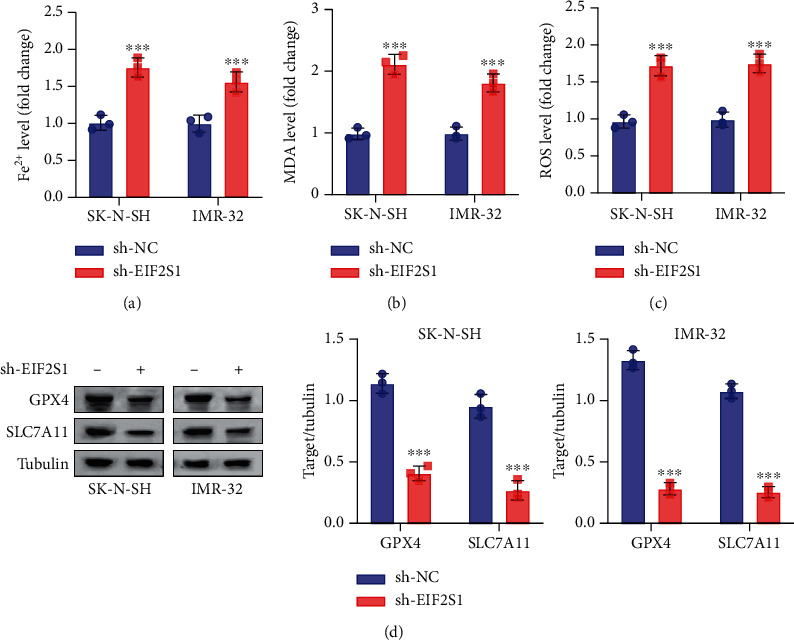
EIF2S1 silencing increases cellular ROS and induces ferroptosis features in neuroblastoma cells. (a) Intracellular-free Fe^2+^ levels in SK-N-SH and IMR-32 cells after EIF2S1 silencing. (b) Malondialdehyde (MDA) levels in SK-N-SH and IMR-32 cells after EIF2S1 silencing. (c) Reactive oxygen species (ROS) levels in SK-N-SH and IMR-32 cells after EIF2S1 silencing. (d) Western blot analysis of GPX4 and SLC7A11 protein levels in SK-N-SH and IMR-32 cells after EIF2S1 silencing. *sh-EIF2S1* versus *sh-NC*; ⁣^∗∗∗^ indicates *p* < 0.0001.

We next attempted to examine whether EIF2S1 knockdown affects ferroptosis in NB cells. Examination of labile Fe^2+^ levels in NB cells suggests a significant increase of intracellular free Fe^2+^ after EIF2S1 silencing (Figure 8(a), *p* < 0.0001). Given that increased levels of ROS and MDA are distinct characteristics of ferroptosis, we examined these markers after silencing EIF2S1. EIF2S1 knockdown induced significant increases in both ROS and MDA levels in NB cells (Figures 8(b) and 8(c), *p* < 0.0001). Western blot analysis of GPX4 and SLC7A11, two critical suppressors of ferroptosis, demonstrated that EIF2S1 silencing resulted in significant reductions of GPX4 and SLC7A11 protein levels in NB cells (Figure 8(d), *p* < 0.0001). These results collectively indicate that EIF2S1 silencing leads to an increase in intracellular ROS levels and promotes ferroptosis induction in NB cells, which may account for the suppressed cell growth effect after EIF2S1 knockdown.

## 4. Discussion

In the present work, we demonstrated that NB tissues and cell lines exhibit elevated EIF2S1 expression levels, which are associated with an unfavorable prognosis. Our findings reveal the potential diagnostic value of EIF2S1 expression for NB. Functional analysis unveiled the potential roles of EIF2S1 in protein translation processes and the regulation of immune cell infiltration. Additionally, we observed that increased EIF2S1 expression substantially correlates with enhanced immune cell infiltration. Our functional experiments in vitro and in vivo further showed that silencing EIF2S1 leads to reduced NB cell proliferation, impaired migration and invasion, and retarded tumor growth in animal models. Mechanistically, EIF2S1 silencing induced iron overload and elevated ROS levels while downregulating GPX4 and SLCA11. Together, these findings indicate that elevated EIF2S1 expression might protect NB cells from ferroptosis, and targeting EIF2A1 may impede the progression of NB.

Previous reports have suggested ferroptosis as a tumor-suppressive mechanism with therapeutic implications in cancer treatment [9, 17]. The higher intracellular Fe^2+^ overload in NB cells compared to normal cells could contribute to elevated mitochondrial ROS levels and DNA damage, promoting ferroptosis during NB malignancy [18]. Several ferroptosis-inducing agents, such as benzo(a)pyrene-7,8-dihydrodiol-9,10-epoxide [19] and aconitine [3], contribute to the accumulation of ROS levels and thereby display antitumorigenic effects. To identify ferroptosis-related genes involved in the progression of NB, we employed transcriptomic data of NB to mine ferroptosis-related genes. Among them, EIF2S1 was selected for further investigation due to its significant impact on NB prognosis. Our findings revealed that heightened EIF2S1 expression is associated with a worse prognosis in NB patients. Importantly, our in vitro and in vivo experiments demonstrated that EIF2S1 silencing attenuated the aggressiveness of NB cells and resulted in a reduction in tumor burden in nude mice, providing important evidence supporting an oncogenic role of EIF2S1 in NB.

EIF2S1 has been reported to regulate cell proliferation, autophagy, and apoptosis by modulating its downstream factors, such as ATF4 and ERK1/2 [20, 21]. However, its regulatory role in ferroptosis sensitivity remained unknown. Our investigation revealed a significant increase in Fe^2+^ levels upon EIF2S1 silencing. The elevated Fe^2+^ levels observed following EIF2S1 silencing are particularly important in the context of the Fenton reaction, where Fe^2+^ catalyzes H₂O₂ to generate toxic hydroxyl radicals (•OH), initiating cellular lipid peroxidation [22, 23]. The resulting intracellular ROS can trigger a series of events in cell membranes, leading to lipid peroxidation and the production of MDA, a primary byproduct of lipid peroxidation. Therefore, our findings indicate that EIF2S1 is essential for the maintenance of iron homeostasis and redox balance in NB cells.

Accumulating evidence suggests that cancer cells tend to maintain a higher level of ROS and Fe^2+^ iron due to metabolic reprogramming, which can significantly impinge on ferroptosis sensitivity [17, 24–26]. Our findings demonstrated that silencing EIF2S1 in NB cells generates increased Fe^2+^ levels alongside elevated MDA and ROS levels. This is consistent with the previous observation that suppressing EIF2S1 activity leads to reduced GSH levels and impaired ROS scavenging in breast cancer [20]. Importantly, we also observed that EIF2S1 silencing results in a reduction of GPX4 and SLC7A11 levels. GPX4 is a pivotal selenoenzyme to protect against ferroptosis. Its catalytic activity effectively diminishes lipid peroxide levels by using GSH, thereby suppressing ferroptosis [27]. SLC7A11 is a member of the cystine transporter complex which imports cystine for GSH biosynthesis [28]. Therefore, our data indicate that the induction of ferroptosis features by EIF2S1 knockdown may be attributed to suppressed expression of GPX4 and SLC7A11 levels. Nevertheless, the mechanisms by which EIF2S1 regulates the expression of GPX4 and SLC7A11 warrant future investigation.

The identification of EIF2S1 as a potential molecular target for NB may open up new avenues for therapeutic intervention. Our findings suggest that targeting EIF2S1 could be a promising strategy for NB treatment. Potential approaches could include the development of small molecule inhibitors or siRNA-based therapies specifically targeting EIF2S1. By suppressing EIF2S1 expression or activity, we may be able to induce ferroptosis in NB cells, thereby inhibiting tumor growth and metastasis. Furthermore, EIF2S1 expression levels could serve as a prognostic biomarker, helping to stratify patients and guide treatment decisions. Combination therapies that pair EIF2S1 inhibition with existing chemotherapeutic agents might also enhance treatment efficacy. Future clinical studies will be crucial to validate these potential applications and translate our findings into improved outcomes for NB patients.

Our study, while revealing the significant role of EIF2S1 in NB progression, has certain limitations that warrant further investigation. Notably, we did not explore the mechanisms underlying EIF2S1 overexpression in NB. Given that N-Myc gene amplification is a common event and a strong indicator of unfavorable NB prognosis [29–32], it is crucial to investigate the potential functional interaction between N-Myc and EIF2S1 during NB development and progression. N-Myc, as a transcription factor that is frequently upregulated in NB [29–32], might be driving the overexpression of EIF2S1 in NB cells. Future studies should examine this hypothesis by analyzing the N-Myc binding sites in the EIF2S1 promoter region and conducting chromatin immunoprecipitation (ChIP) assays. Additionally, collecting clinical samples to assess the expression correlation between N-Myc and EIF2S1 through RT-qPCR and immunohistochemistry would provide valuable insights into their relationship in NB patients. Furthermore, exploring other potential transcriptional regulators and signaling pathways involved in EIF2S1 overexpression would contribute to a more comprehensive understanding of NB pathogenesis and potentially reveal additional therapeutic targets. Moreover, our findings on immune infiltration in NB highlight the need for further investigation into the complex interplay between EIF2S1 expression, immune cell populations, and tumor microenvironment. Future studies should aim to elucidate the mechanistic links between EIF2S1 and immune regulation in NB, potentially uncovering new avenues for combination therapies that target both EIF2S1 and immune-related pathways.

## 5. Conclusion

In summary, our study demonstrates that EIF2S1 is involved in regulating NB cell ferroptosis through modulation of GPX4 and SLC7A11. EIF2S1 silencing results in reduced cell proliferation and migration abilities of NB cell lines, as well as impaired tumorigenicity in vivo. EIF2S1 knockdown serves as a potent inducer of ferroptosis in NB cells, characterized by elevated intracellular Fe^2+^ levels and augmented ROS production, with the concomitant downregulation of GPX4 and SLC7A11. Our findings provide new insights into the molecular mechanisms underlying NB progression and highlight EIF2S1 as a potential therapeutic target. Future research should focus on developing strategies to modulate the EIF2S1/GPX4 axis as a novel approach to combat NB.

## Figures and Tables

**Table 1:** The correlation between candidate genes and disease prognosis.

**Gene**	**KM**	**HR**	**HR. 95**L	**HR. 95H**	**Cox ** **p** ** value**
AKR1C1	0.000281898	0.529683363	0.393481604	0.713030704	2.79e − 05
EIF2S1	0.000308807	3.055359761	1.645003342	5.674896232	0.000406864
FTH1	2.08e − 05	1.515031339	1.241990548	1.848097765	4.18e − 05
HSF1	2.03e − 06	2.96588726	1.746990899	5.035222133	5.68e − 05
LONP1	1.41e − 05	2.72985566	1.696166196	4.393503386	3.53e − 05
ULK2	0.000441834	0.487598186	0.337255578	0.704960887	0.00013415

**Table 2:** The correlation between EIF2S1 expression and clinical features in 36 NB patients.

**Characteristics**	**No.**	**EIF2S1 expression**		**p** ** value**
		**Low (** **n** ** = 18)**	**High (** **n** ** = 18)**	
Gender				0.18
Male	20	8	12	
Female	16	10	6	
Age				0.278
≥ 18 months	25	11	14	
< 18 months	11	7	4	
Risk group				0.04
Low+intermediate	22	14	8	
High	14	4	10	
MYCN status				0.287
Amplified	8	3	5	
Nonamplified	26	13	13	
Not available	2	2	0	
Stage				0.019
I+II+IVs	17	12	5	
III+IV	19	6	13	
Pathological subtype				0.298
NB+GNBn	23	10	13	
GNBi+GN	13	8	5	
Distant metastasis				0.044
Yes	16	5	11	
No	20	13	7	

## Data Availability

The datasets used and/or analyzed during the current study are available from the corresponding author via email request.

## Nomenclature

BCA: bicinchoninic acid
cDNA: complementary DNA
ECL: enhanced chemiluminescence
enrichGO: enrichment analysis
FBS: fetal bovine serum
GSH: glutathione
HUVECs: human umbilical vein endothelial cells
INSS: International Neuroblastoma Staging System
KEGG: Kyoto Encyclopedia of Genes and Genomes
MDA: malondialdehyde
MOI: multiplicity of infection
NB: neuroblastoma
PVDF: polyvinylidene fluoride
RIP: ribonucleoprotein immunoprecipitation
ROS: reactive oxygen species
RT-qPCR: reverse transcriptase-polymerase chain reaction
shRNAs: short hairpin ribose nucleic acids

## Supporting Information

Supporting Information 1 Table S1. Genes significantly associated with EIF2S1 expression.

Supporting Information 2 Figure S1. Pearson's analysis reveals the correlation of EIF2S1 expression with critical genes in NB samples.

Supporting Information 3 Figure S2. The heat map shows differentially expressed genes in EIF2S1-high and EIF2S1-low groups.
