# Supplementary figures and images for: EIF2S1 Silencing Impedes Neuroblastoma Development Through GPX4 Inactivation and Ferroptosis Induction
Source: Int J Genomics. 2024 Oct 19;2024:6594426. doi: 10.1155/2024/6594426 (PMC11512646; doi:10.1155/2024/6594426)

## Slide 1
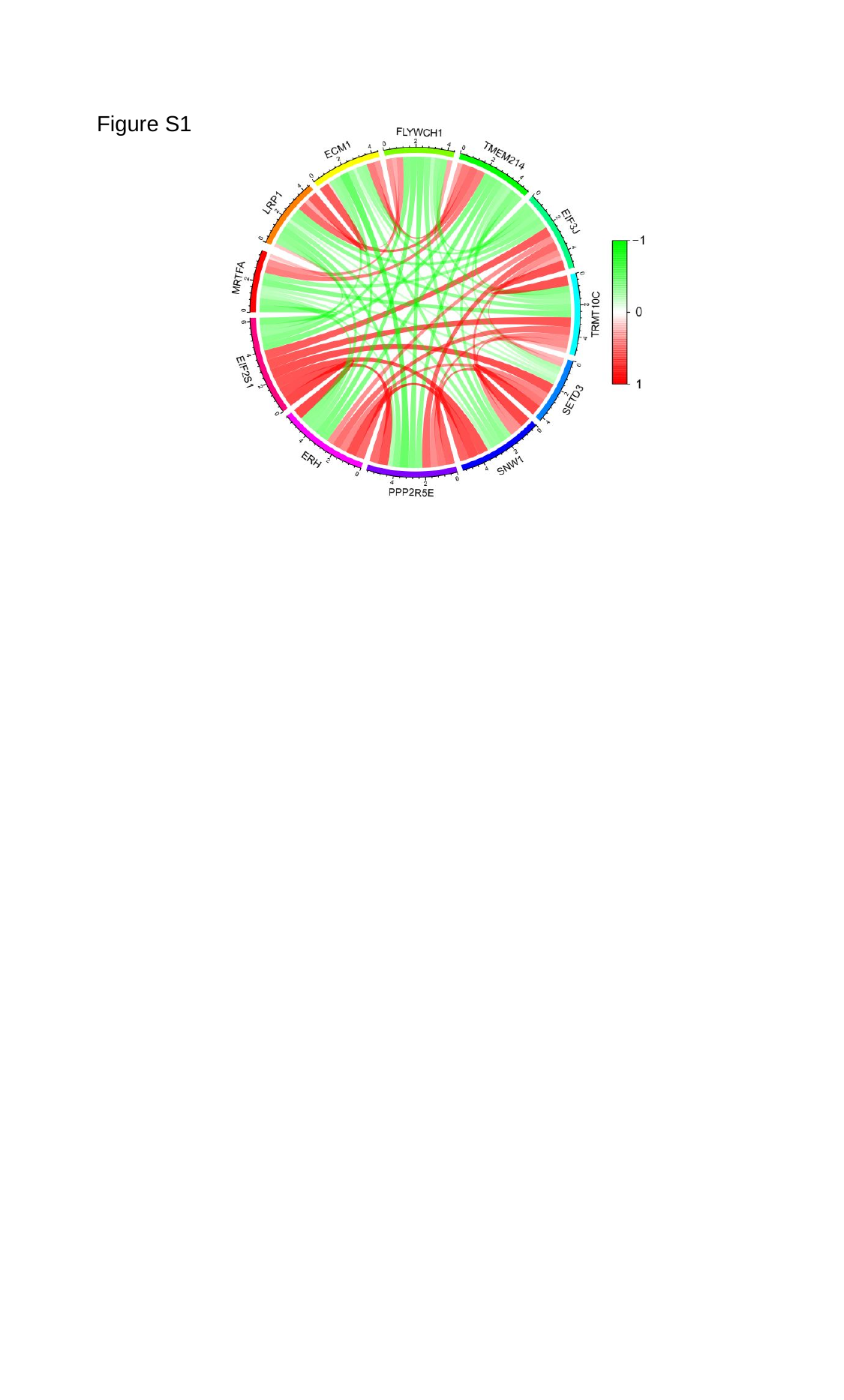

Figure S1

Supplement: Supporting Information 2 — Figure S1. Pearson's analysis reveals the correlation of EIF2S1 expression with critical genes in NB samples. [file 6594426.f2.pptx]

## Slide 1
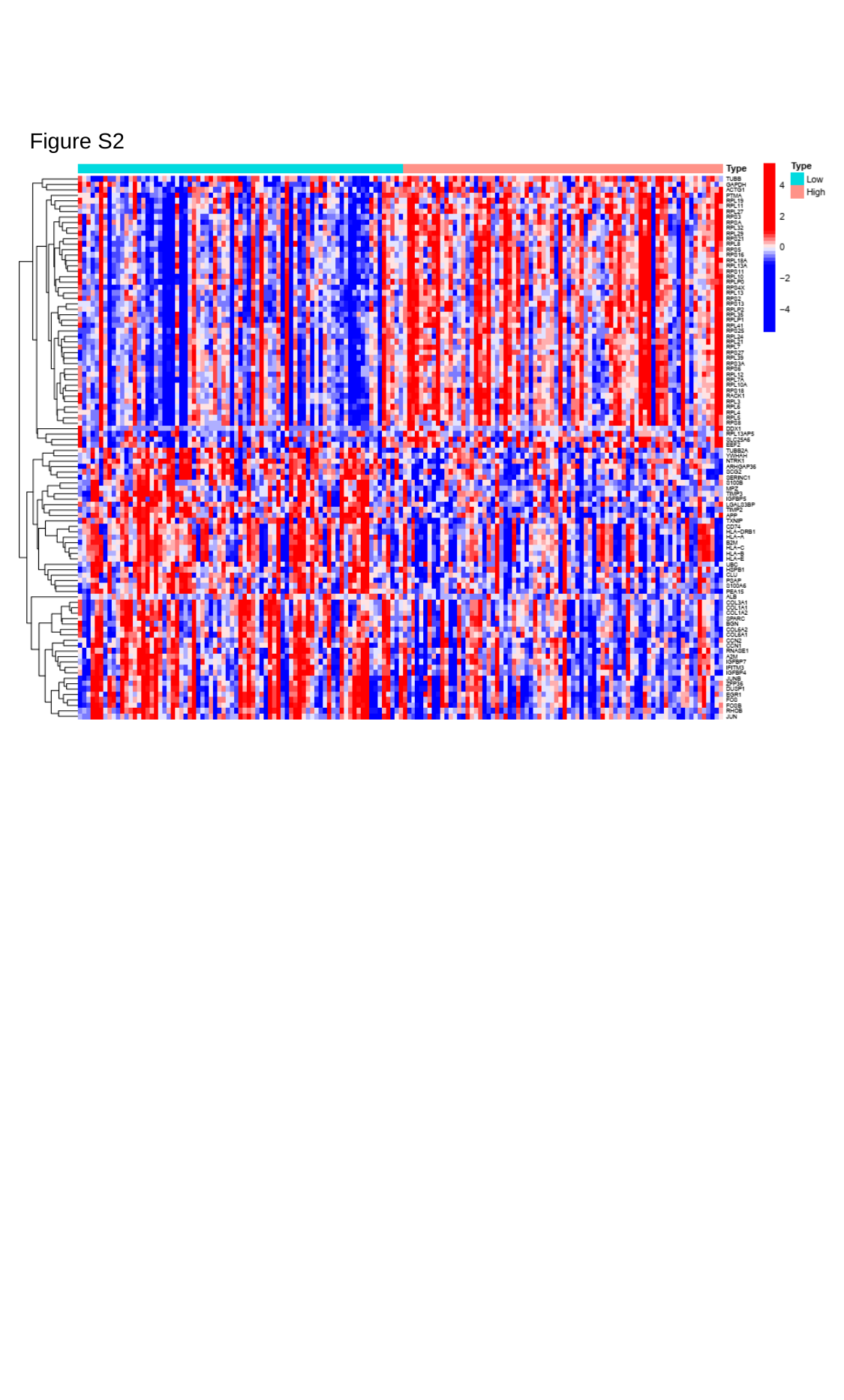

Figure S2

Supplement: Supporting Information 3 — Figure S2. The heat map shows differentially expressed genes in EIF2S1-high and EIF2S1-low groups. [file 6594426.f3.pptx]
